# *N*-acetyl-L-cysteine ameliorates the inflammatory disease process in experimental autoimmune encephalomyelitis in Lewis rats

**DOI:** 10.1186/1740-2557-2-4

**Published:** 2005-05-03

**Authors:** Romesh Stanislaus, Anne G Gilg, Avtar K Singh, Inderjit Singh

**Affiliations:** 1 Department of Biostatistics, Bioinformatics & Epidemiology, Medical University of South Carolina, Charleston, SC, USA; 2 Department of Pediatrics, Medical University of South Carolina, Charleston, SC, USA

**Keywords:** EAE, Macrophages, infiltration *N*-acetyl-L-cysteine, CNS

## Abstract

We report that *N*-acetyl-L-cysteine (NAC) treatment blocked induction of TNF-α, IL-1β, IFN-γ and iNOS in the CNS and attenuated clinical disease in the myelin basic protein induced model of experimental allergic encephalomyelitis (EAE) in Lewis rats. Infiltration of mononuclear cells into the CNS and induction of inflammatory cytokines and iNOS in multiple sclerosis (MS) and EAE have been implicated in subsequent disease progression and pathogenesis. To understand the mechanism of efficacy of NAC against EAE, we examined its effect on the production of cytokines and the infiltration of inflammatory cells into the CNS. NAC treatment attenuated the transmigration of mononuclear cells thereby lessening the neuroinflammatory disease. Splenocytes from NAC-treated EAE animals showed reduced IFN-γ production, a Th1 cytokine and increased IL-10 production, an anti-inflammatory cytokine. Further, splenocytes from NAC-treated EAE animals also showed decreased nitrite production when stimulated *in vitro *by LPS. These observations indicate that NAC treatment may be of therapeutic value in MS against the inflammatory disease process associated with the infiltration of activated mononuclear cells into the CNS.

## 1. Introduction

Multiple sclerosis (MS) is a chronic demyelinating disease marked by focal destruction of myelin, resulting in the loss of oligodendrocytes and axons accompanied by an inflammatory disease process [1-3]. Experimental autoimmune encephalomyelitis (EAE) is an animal model of MS. Both MS and EAE are initiated by a T-cell mediated autoimmune response (CD4+ and CD8+) against myelin components followed by induction of inflammatory mediators (chemokines and cytokines) that in turn define the pattern of perivascular migration of activated T-cells and mononuclear cells into the CNS [1-4].

The sequence of events associated with loss of oligodendrocytes and myelin in MS and EAE are not precisely understood. A complex interaction between the mediators released by infiltrating cells and brain endogenous activated glial cells (astrocytes and microglia) are believed to contribute towards the inflammatory disease process and tissue damage [1-3,5-7]. Numerous studies have documented the expression of proinflammatory cytokines (TNF-α, IL-1β, and IFN-γ) in EAE and MS tissue and increased levels of IFN-γ and TNF-α levels in CNS or plasma appear to predict relapse in MS [1-3,8]. On the other hand, enhanced expression of anti-inflammatory cytokines (IL-4, IL-10 and TGF-β) appears to mediate disease remission [1-3,9]. In MS brain, expression of iNOS by activated astrocytes, microglia and macrophages is associated with the demyelinating regions [10-13]. The NO derived from iNOS as ONOO^- ^(a reaction product of NO and O_2_^-^) is thought to play a role in the pathobiology of MS and EAE. Peroxynitrite (ONOO^-^) is able to modify proteins, lipids and DNA resulting in damage to oligodendrocytes and myelin [1-3].

In spite of extensive research to develop pharmacotherapeutic agents to ameliorate or reduce the number of exacerbations and subsequent progression of neurological disability in MS, only a few therapies are available. Presently, IFN-β [14] and glatiramer acetate [15] are used in treatment of MS but the therapeutic efficacy of these compounds is limited by significant side effects. Recent studies from our laboratory [16,17] and others [18] report the potential of HMG-CoA reductase inhibitors (statins) in attenuating the disease process in EAE. The efficacy derives from a shift from an inflammatory Th1 response towards an anti-inflammatory Th2-biased response [16,18,19], blocked infiltration of mononuclear cells into CNS [20] and attenuation of the induction of proinflammatory cytokines (TNF-α, IFN-γ) and iNOS in the CNS of EAE animals [17,20].

Reactive oxygen species (ROS) and reactive nitrogen species (RNS), generated as a result of the inflammatory process, are believed to play a role in the pathobiology of EAE and MS [10,12,13]. Cell culture studies showed that NAC, a potent antioxidant, inhibited induction of TNF-α and iNOS and NO production in peritoneal macrophages, C6 glial cells and primary astrocytes, and blocked the activation of NFκB in peritoneal macrophages [21]. Accordingly, oral administration of the oxidant scavenger NAC was found to attenuate EAE clinical disease [22]. The present studies were designed to elucidate the mechanism of observed therapeutic efficacy of NAC against EAE. These studies document that NAC treatment inhibited the clinical disease by attenuating multiple events in EAE disease such as shifting the immune response from a Th1 bias, increasing IL-10 cytokine production by splenocytes, attenuating transmigration of mononuclear cells, and inhibiting induction of proinflammatory cytokines (TNF-α, IL-1β, IFN-γ) and iNOS in the CNS. Taken together these results suggest NAC may be of therapeutic value for cell-mediated autoimmune diseases such as multiple sclerosis.

## 2. Materials and methods

### Chemicals

Myelin basic protein (MBP) isolated from guinea pig brain and complete Freund's adjuvant (CFA) and pertussis toxin were obtained from Sigma (St. Louis, MO). *N*-acetyl-L-cysteine (NAC) was obtained from Calbiochem (USA).

### EAE induction and treatment with NAC in Lewis rats

Experiments were performed on female Lewis rats (Harlan Laboratory, USA) weighing 250–300 g. Animals were housed in the animal care facility of the Medical University of South Carolina, USA, throughout the experiment and provided with food and water *ad libitum*. All experimental protocols were reviewed and approved by the Institutional Animal Care and Use Committee. EAE was induced by subcutaneous injection of 50 μg of MBP (per animal) emulsified in complete Freund's adjuvant in the region of the footpad of the hind leg on day 1 followed by a booster injection of the same on day 7. Additionally, animals received 200 ng of pertussis toxin on days 0 and 1. Clinical signs in these rats manifest as ascending paralysis resulting in EAE in most animals. The clinical signs of EAE were scored by a masked investigator as 0 = normal; 1 = piloerection; 2 = loss in tail tonicity; 3 = hind leg paralysis; 4 = paraplegia; and 5 = moribund. NAC treatment was started on the first day of immunization (day 1) and continued daily for the duration of the experiment. One group of rats induced for EAE (n = 15) was given intraperitoneal injections of NAC (150 mg/kg body weight in PBS with pH adjusted to 7.2 with NaOH). The second group of rats (n = 15) was induced for EAE and treated with the vehicle (PBS). Animals receiving only CFA were used as the control group (n = 15). Untreated EAE animals were sacrificed at clinical stage 4 (paraplegia) or 5 (moribund) according to approved protocol. NAC treated animal group was sacrificed at their peak clinical disease, which was an average clinical score of 3, as determined from preliminary studies. Tissue for histology and immunohistochemistry and splenocytes were recovered for analysis.

### Histopathology-Immunohistochemistry

The lumbar region of the spinal cord was dissected and carefully processed for histological and immunohistological examination (n = 12). Spinal cords were fixed in 10% buffered formalin (Stephens Scientific, Riverdale, NJ), embedded in paraffin and sectioned at 4 μm thickness. Sections were then stained for various cytokines and cell markers.

Immunohistochemistry for TNF-α, IFN-γ, IL-1β, iNOS and nitrotyrosine was done as previously described [17]. Sections were incubated with appropriate antibodies (1:100) overnight followed by fluorochrome conjugated secondary IgG antibody (1:100, Sigma, St. Louis, MO) and mounted with Fluoromount G (EMS, Fort Washington, PA). Non-immune IgG was used as control primary antibody. Sections were also incubated with TRITC or FITC conjugated IgG without the primary antibody as negative control. Nuclear staining was performed using DAPI (Sigma, St. Louis, MO) and hematoxylin and eosin (H&E) staining was performed as described by Kiernan, J.A (1990). All the sections were analyzed using an Olympus microscope (Olympus BX60, Opelco, Dulles, VA) and images were captured using a digital video camera (Olympus U-CMAD-2, Optronics, Galeta, CA) and Adobe Photoshop (Adobe Systems, CA).

### Quantitative analysis of infiltrating cells

Infiltrating cells labeled with either ED1 or DAPI were analyzed using Image-Pro Plus 4.0 (Media Cybernetics, Maryland, USA) software. Individual sections were analyzed and the mean and SD were calculated for each group (n = 12). The group means were compared and the significance of difference was determined. A p value of <0.05 was considered significant. This analysis was done using the Regression Data Analysis tool of Microsoft Excel 4.0 (Microsoft, Redmount, WA).

### Splenocyte Isolation and Cell Culture

Splenocytes were isolated from each animal group (Control, EAE, EAE+NAC) (n = 6) using Lympholyte^®^-Rat (Cedarlane Laboratories Ltd., Hornby, Canada) density separation medium according to manufacturer's instruction. The cell concentration in the suspension was adjusted to 2 × 10^7 ^nucleated cells per ml or less, layered on Lympholyte^®^-Rat density separation medium, and centrifuged for 20 min at 1000 g – 1500 g at room temperature. The interface formed after the centrifugation was then extracted using a Pasteur pipette, and transferred to a new centrifuge tube. The transferred cells were then diluted with medium, and centrifuged at 800 g for 10 min, washed twice with media, and cultured in 24-well plates at a concentration of 5 × 10^6 ^cells/ml. The cells were then stimulated *in vitro *with MBP (20 μg/mL), LPS (1 μg/mL), or PHA (10 μg/mL), (Sigma, St. Louis, MO, USA), or without any stimulants for 48 hrs. Each treatment was performed in triplicate. At the end of the 48 hr. incubation period, supernatants were collected and used for the measurement of cytokines and nitrite.

### ELISA

Cytokines (IFN-γ and IL-10) were detected in culture supernatants using commercially available OptEIA™ kits from PharMingen (San Diego, CA, USA) according to manufacturer's instructions. The assay procedure is as follows: 96-well microplates were coated with capture antibody diluted in coating buffer overnight at 4°C. Plates were then washed and blocked with assay diluent (PharMingen, San Diego, CA, USA) for 1 hr at room temperature. Blocked plates were then washed, and the standards and samples added to the wells and incubated for 2 hr. at room temperature. At the end of incubation, plates were washed and working detector (detection antibody + Avidin-HRP) was added to the wells and incubated for 1 hr. at room temperature. Following incubation, plates were washed and TMB substrate reagent was added (PharMingen, San Diego, CA, USA) to the wells for 30 min. at room temperature in the dark. At the end of the incubation, stop solution (1 M H3PO4) was added, and absorbance read at 450 nm using a Spectramax^® ^microplate spectrophotometer (Molecular Devices, Sunnyvale, CA, USA).

### Nitrite measurement

Nitrite levels were determined on isolated splenocytes with Griess reagent as previously described [23] with minor modifications. One hundred μl of culture supernatant was allowed to react with 100 μl of Griess reagent and incubated at room temperature for 15 min. The optical density of the assay samples was measured at 570 nm using a 96-well plate Spectramax^® ^microplate reader with SOFTMAX^® ^software (Molecular Devices, Sunnyvale, CA, USA). Fresh culture media served as the blank in all experiments. Nitrite concentrations were calculated from a standard curve derived from the reaction of NaNO_2 _in the assay.

## 3. Results

### Effect of NAC on the Clinical Signs of Rats

**Figure 1:**
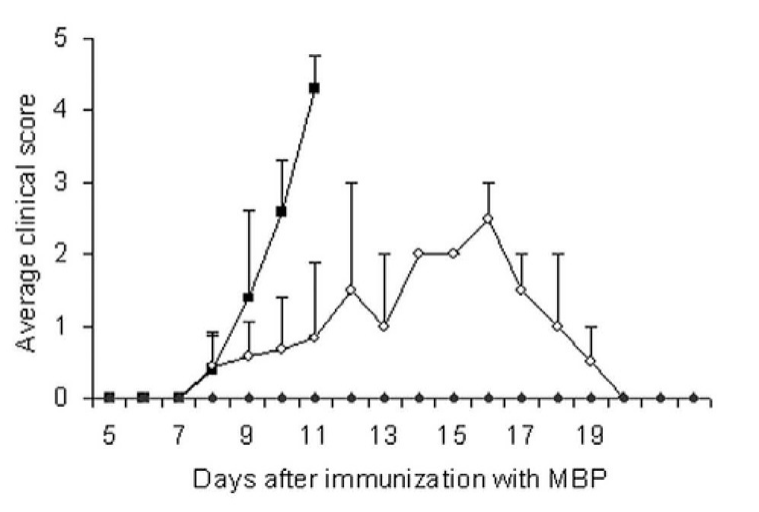
The protective effect of NAC on the clinical signs of MBP induced EAE in female Lewis rats. EAE was induced as described in Materials and Methods. Data are given as average clinical disease score where 0 = normal; 1 piloerection; 2 = loss in tail tonicity; 3 = hind leg paralysis; 4 = paraplegia and 5 = moribund. Each group i.e., MBP (closed squares), MBP + NAC (open circles) treated and control group (closed diamonds) had 15 animals (n = 15). EAE animals reached a peak clinical score of 4 or 5 on or around the 11^th ^day after first immunization and were sacrificed according to approved protocol. NAC treated animals had milder clinical signs (average clinical score of 3 as compared to 4 or 5 for EAE). Control group did not show any clinical symptoms (clinical score = 0).

Our goal was to investigate the effect of NAC on rats induced for acute EAE. In the Lewis rat model MBP induces an acute monophasic disease progression. As shown in Fig. 1, clinical signs of EAE were evident in MBP-treated Lewis female rats from the 8^th ^day after first immunization inducing an acute monophasic disease progression resulting in paraplegia (clinical score of 4) or moribund state (clinical score of 5) on or around the 12^th ^day. However, the control animals receiving only complete Freund's adjuvant did not show any disease symptoms (Fig. 1). Animals induced for EAE but given only the vehicle closely followed the disease progression of MBP-treated rats. Treatment of MBP-injected rats with NAC, administered from the first day of immunization, protected the rats by attenuating the severity of disease progression (Fig. 1). NAC treated animals had milder clinical signs (average clinical score of 3 as compared to 5 for EAE).

### Effect of NAC on the infiltration of inflammatory cells into the spinal cord

The neuropathological changes in EAE and MS are associated with the blood brain barrier breakdown and infiltration by mononuclear cells [24,25]. Clinical disease in EAE has been shown to correlate with the invasion of CNS by mononuclear cells. These studies demonstrate that MBP-induced EAE results in the induction of inflammatory disease, and treatment with NAC provides protection against the EAE disease process. Therefore, in order to understand the mechanism of therapeutic efficacy in EAE, we studied the effect of NAC on the invasion of mononuclear cells into the CNS in the EAE model.

**Figure 2:**
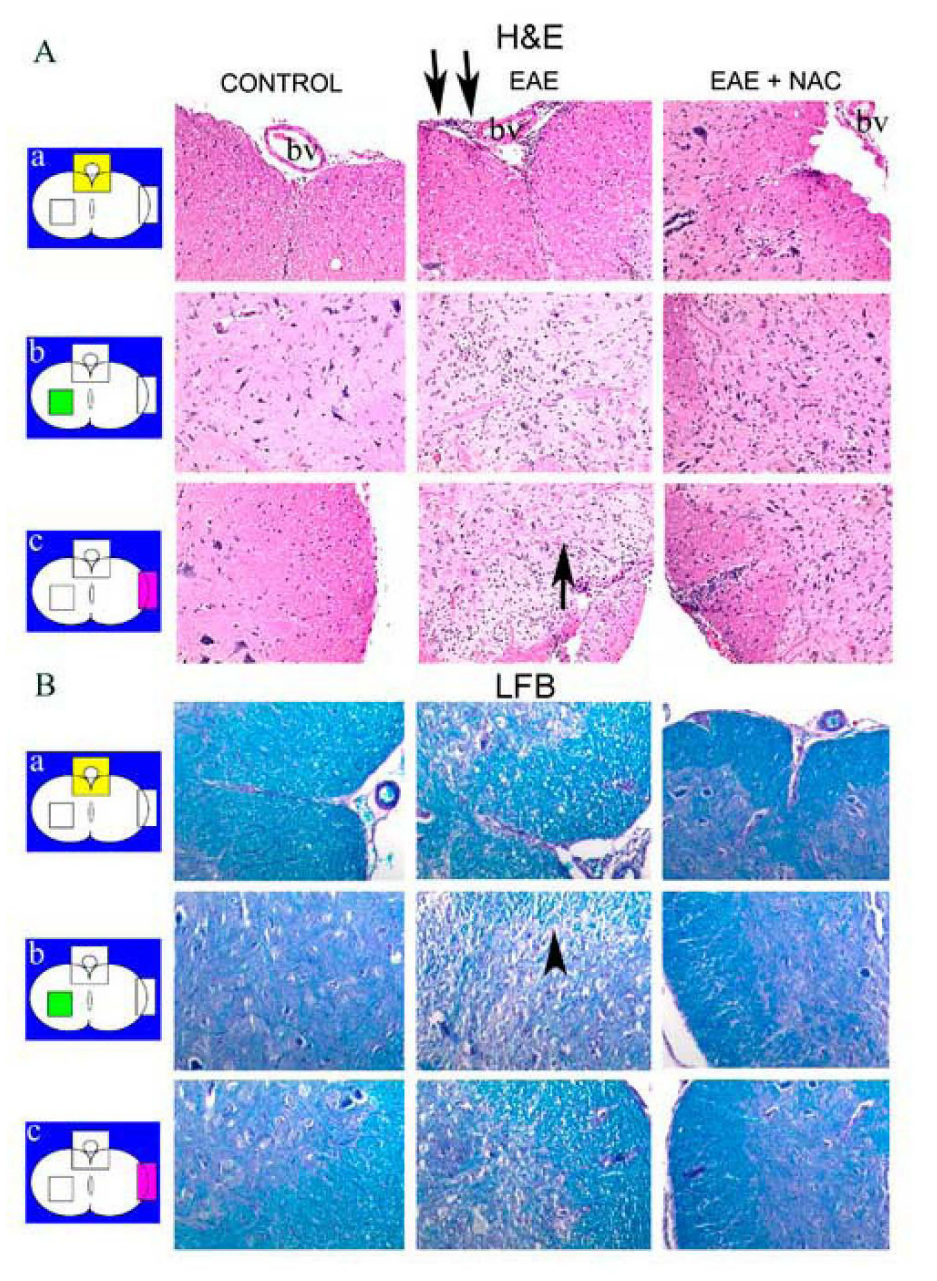
Inflammation and demyelination in sections of lumbar spinal cord from control, EAE and EAE + NAC (n = 12) treated Lewis rats. The spinal cords were isolated at peak manifestation of the disease (i.e. clinical score 5 in EAE and 3 in EAE + NAC treated animals). Photomicrographs represent regions from a) anterior cord b) central region and c) lateral cord. BV-denotes blood vessel. A. H&E staining of cross-sections of lumbar spinal cord. Compared to the control group, Lewis rats with EAE demonstrated gliosis (single arrow) and perivascular (double arrows), meningeal and interstitial chronic inflammatory infiltrates. These effects were attenuated in sections from EAE+NAC treated animals. B. LFB-PAS staining of cross sections of lumbar spinal cord from control, EAE, and EAE+NAC treated Lewis rats. Compared to the control animals, the interface of normal to demyelinating plaque (arrowhead) is notable in sections from the EAE group of animals. Myelin persists in the plaque as globules in the cytoplasm of macrophages. The EAE+NAC group showed demyelination, but to a lesser degree than that seen in the untreated group.

**Figure 3:**
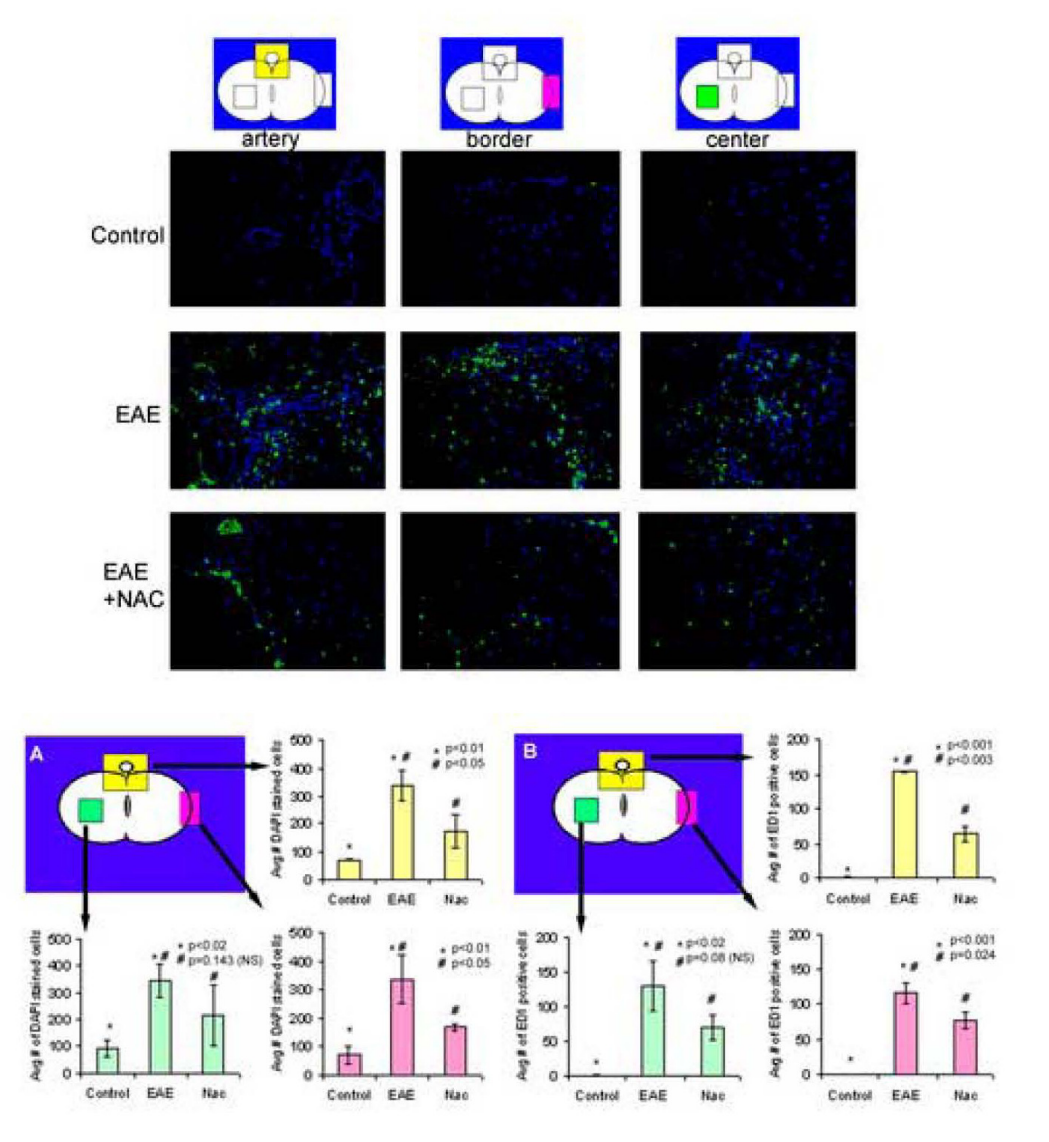
Quantification of the inflammatory infiltrates by immunostaining of Lewis rat spinal cord (n = 12). Top panel: The spinal cords were isolated when the animals were showing maximum clinical symptoms (i.e. for the EAE group clinical score of 5 and EAE+NAC clinical score of 3). The sections were stained for ED1 (macrophage/monocyte -green) and nuclei labeled with DAPI (blue) as described in materials and methods. Spinal cords of rats induced for EAE demonstrated increased numbers of ED1 positive cells (green) and other glial and inflammatory cells (blue) in the CNS. Original magnification 200×. Bottom panel: Quantification of the infiltrates showed significant numbers of glial and inflammatory cells (A: DAPI; nuclei stained blue) of which many also were positive for macrophage/monocyte (B: ED1; stained green) in the spinal cord of EAE animals as compared to control and EAE + NAC treated animals.

The spinal cords of rats induced for EAE had heavy mononuclear inflammatory infiltrates on the meningeal surfaces, perivascular areas and interstitial areas as seen by H&E staining (Fig. 2a). EAE animals treated with NAC showed infiltration of the CNS by inflammatory cells but not to the extent as that seen in EAE animals. Further analysis of the cell infiltrates was performed to identify the major cell type infiltrating the CNS in addition to the T-cells. Immunohistochemical methods using ED1 (monocyte/ macrophage marker) and DAPI (for nucleated cells) were performed. As seen in Fig. 3, EAE animals showed the most infiltration by ED1 positive cells. In contrast the NAC-treated animals showed significantly less infiltration by ED1 positive cells (reduced by an average of 46 percent). Quantitative analysis of cell infiltrates into the CNS showed a significant amount of nucleated as well as ED1 positive cells in the CNS of EAE animals (Fig. 3b). In contrast, cell infiltration into the CNS of treated animals was significantly less than that seen in EAE animals (reduced by an average of 45 percent).

### Effect of NAC on the expression of pro-inflammatory cytokines and iNOS in the spinal cord

**Figure 4:**
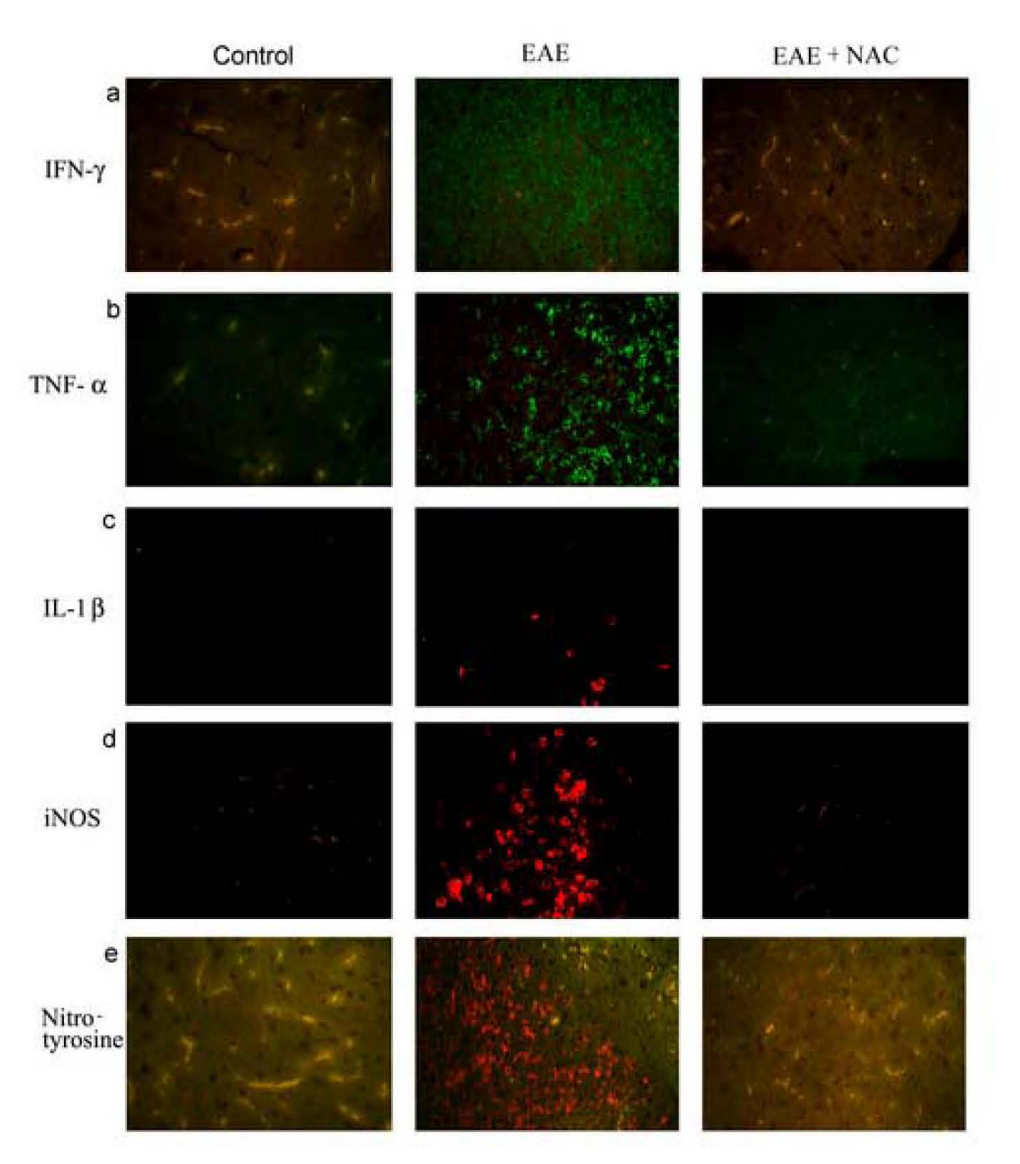
Immunofluorescent detection of IFN-γ, TNF-α, IL-1β, iNOS and nitrotyrosine in the CNS of female Lewis rats. The spinal cords were isolated when the animals were showing maximum clinical symptoms (i.e. for the EAE group clinical score of 5 and EAE+NAC clinical score of 3). Immunostaining was performed in spinal cord sections (n = 12) of female Lewis rats as described in Materials and Methods. EAE sections showed intense staining for IFN-γ, TNF-α, iNOS and nitrotyrosine with less intense staining for IL-1 β. Control and EAE + NAC sections showed minimal staining (original magnification 200×).

Since the major source of IL-1β in EAE is monocytes/macrophages, as further evidence for macrophage infiltration we examined the expression of IL-1β in the CNS. As evidenced by Fig. 4c, expression of IL-1β was evident in the CNS of EAE induced animals and to a far lesser degree in the NAC treated animals. IL-1β expression was also co-localized to ED1 positive cells in EAE animal spinal cords (data not shown). We also examined the expression of proinflammatory cytokines (TNF-α and IFN-γ), iNOS and nitrotyrosine in the spinal cord sections from control, EAE, and NAC-treated EAE rats using immunohistochemistry. As seen in figure 4a–e, MBP-induced EAE resulted in the expression of TNF-α, IFN-γ, IL-1β, IFN-γ, iNOS and nitrotyrosine. NAC treatment of EAE blocked the induction of these cytokines, iNOS, and nitrotyrosine similar to control animals.

### IFN-γ, IL-10 and nitrite production by splenocytes from EAE and treated animals

**Figure 5:**
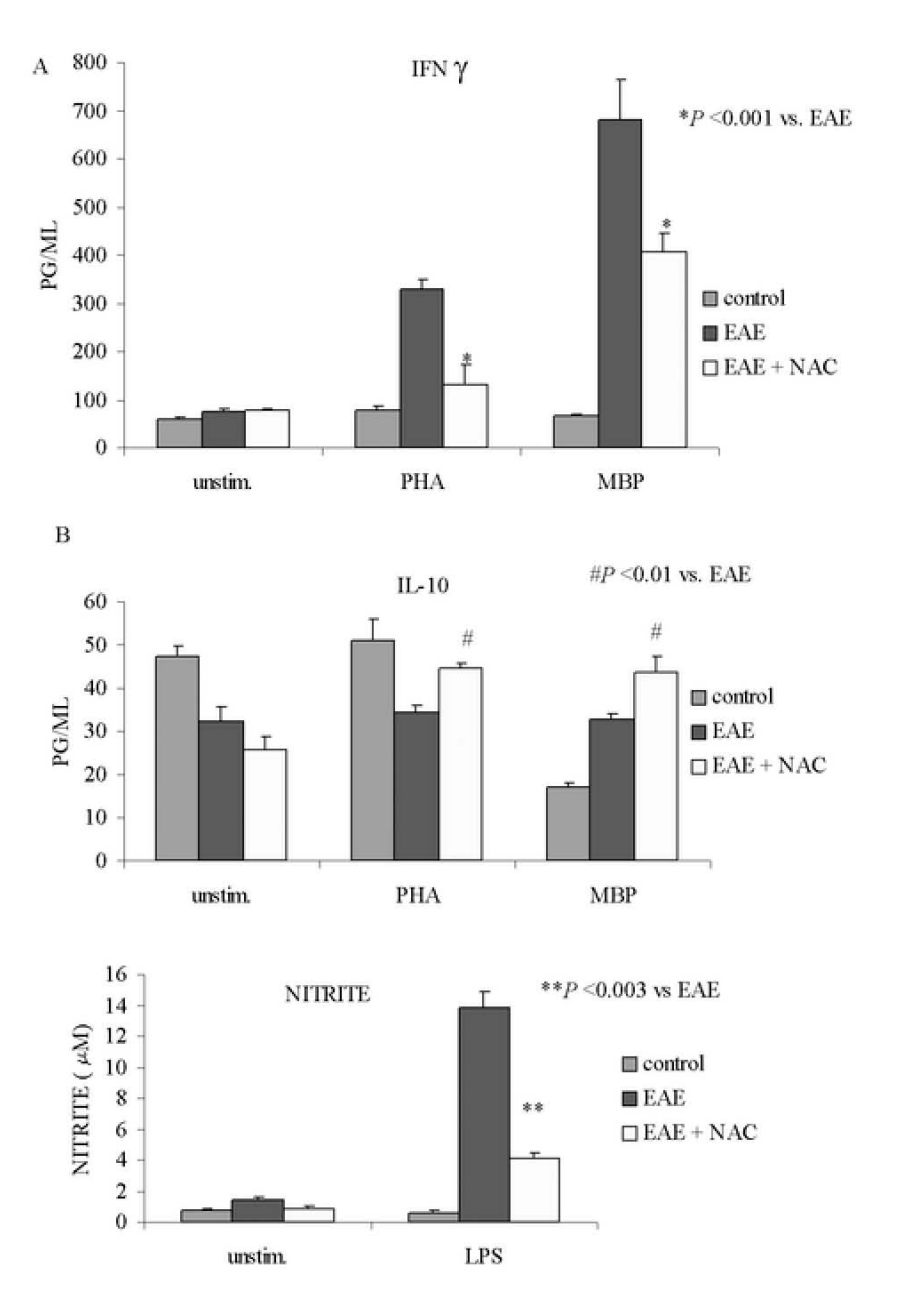
IFN-γ, IL-10 and nitrite production by splenocytes from control, EAE and treated animals. Splenocytes (8 × 10^5 ^cells per well) were obtained from Control, EAE, and EAE + NAC treated rats when they were showing maximum clinical signs, and were stimulated *in vitro *with PHA (10 μg/ml, a & b), MBP (20 μg/ml, a & b) or LPS (1 μg/ml, c) for 48 hrs. Each treatment was performed in triplicate for each animal group (n = 6). a and b: The levels (pg/ml) of IFN-γ and IL-10 in culture supernatants were measured using ELISA kits. There was a significant increase in IFN-γ and decrease in IL-10 in splenocytes from untreated EAE animals stimulated with PHA and MBP. EAE + NAC splenocytes showed reduced IFN-γ production whereas IL-10 production was increased. c: Culture supernatants were collected and accumulated nitrite, a stable product of NO production, was measured using Griess reagent. LPS-stimulated EAE splenocytes showed significantly higher levels of nitrite as compared to control and this was reduced with NAC treatment.

In vitro splenocytes assays were performed to elucidate whether NAC treatment could cause a shift to Th2-type T-cell activity. In order to examine this effect, we studied the effect of NAC on the major Th2 cytokine in the EAE disease process, IL-10. Splenocytes (8 × 10^5 ^cells per well) were obtained from Control, EAE, and EAE + NAC treated rats. Cells were stimulated *in vitro *with PHA (10 μg/ml, a & b), MBP (20 μg/ml, a & b) or LPS (1 μg/ml, c) for 48 hrs. The levels (pg/ml) of IFN-γ and IL-10 in culture supernatants were measured using ELISA kits. As seen in Fig. 5 there was a significant increase in IFN-γ (5a) and decrease in IL-10 (5b) in splenocytes from untreated EAE animals. NAC treatment reduced IFN-γ production by splenocytes (by 59% for PHA and 40 % for MBP) and up-regulated IL-10 production by EAE splenocytes (by 31% for PHA and 34% for MBP). Culture supernatants were collected and accumulated nitrite, a stable product of NO production, was measured using Griess reagent. NAC treatment also inhibited the production of nitrite by LPS-stimulated splenocytes by 71% as compared to splenocytes from EAE animals. These studies indicate that NAC treatment reduced IFN-γ, a proinflammatory Th1 cytokine and increased IL-10, an anti-inflammatory cytokine.

## 4. Discussion

The evidence presented in this paper demonstrates that NAC treatment reduced the inflammatory monocyte/macrophage cells in the CNS of Lewis rats with acute monophasic EAE. This in turn results in protection both in terms of clinical and histopathological changes. These conclusions are based on the following observations. 1) NAC treatment of EAE rats reduced the severity of EAE clinical symptoms, 2) attenuated the infiltration of mononuclear cells into the CNS of EAE rats, 3) blocked the induction of proinflammatory cytokines, iNOS and nitrotyrosine in the CNS, and 4) decreased proinflammatory Th1 cytokine responses (IFN-γ) from *ex vivo *splenocytes while increasing anti-inflammatory cytokine production (IL-10), and decreasing NO production in LPS-stimulated splenocytes.

The infiltration of activated mononuclear cells into the CNS of EAE is a critical event in the progression of the disease [26]. We have shown both qualitatively and quantitatively that ED1 positive leukocytes, namely macrophage/monocytes, were significantly decreased in animals treated with NAC as compared to the EAE animals. This decrease also correlated with the amelioration of clinical disease in female Lewis rats. As compared to our previous studies with lovastatin, NAC was not as effective in blocking the transmigration of inflammatory cells (NAC reduced by an average of 46%, while lovastatin reduced by 85%) and hence did not delay the onset of disease as was achieved with lovastatin treatment (EAE, EAE + NAC onset day 8 versus EAE + lovastatin onset day 11). However, NAC reduced the clinical scores to the same levels as those obtained with lovastatin (both had clinical scores maximum of 3). Other studies have also shown a correlation between macrophage infiltration and EAE clinical disease [27]. Inflammatory cytokine expression (IFN-γ, IL-1β, and TNF-α) was also inhibited in the CNS of EAE animals treated with NAC. As a consequence, inhibition of IFN-γ expression in NAC treated animals could in turn result in the reduced expression of MHC II molecules thereby inhibiting the proliferation of T-lymphocytes as has been shown with statins [28,29], copolymer 1 [30] and IFN-β [31].

Evidence indicates that iNOS while not a crucial factor for induction of EAE, plays a major role in the progression of the disease. The critical factors is the amount of NO produced that tips the balance in favor or against the pathogenesis of EAE [32]. The peroxynitrite (ONOO^-^) produced by reaction of NO and O_2_^- ^can damage membranes and cells by nitrosylation of lipids, proteins and nucleic acids. The induction of IL-1β and activation of NFκB were shown to precede the induction of iNOS in ED1^+ ^cells [33]. Here we report that NAC blocked the induction of IL-1β in the CNS of EAE animals. *Ex vivo *studies using splenocytes isolated from control, EAE and EAE+NAC treated animals showed that NAC inhibited IFN-γ production while increasing IL-10 production. These changes coincided with a decreased NO production in the cultured splenocytes. NAC treatment was not as effective as lovastatin in altering cytokine production, but the reduction in nitrite was identical. NAC treatment reduced IFN-γ production by splenocytes (NAC by 59% and 40%, LOV by 76% and 60% for PHA and MBP respectively) and up-regulated IL-10 production by EAE splenocytes (NAC by 31% and 34%, LOV by 350% and 490% for PHA and MBP respectively). NAC treatment also inhibited the production of nitrite by LPS-stimulated splenocytes by 71% as compared to splenocytes from EAE animals. These studies indicate that NAC treatment inhibited a proinflammatory Th1 biased cytokine response (IFN-γ) while promoting an increase in IL-10, an anti-inflammatory cytokine. Similar shifts from Th1 cytokine profile to Th2 have been correlated with disease recovery or improvement in both EAE and MS [16,18,19,34-37].

The brain is particularly vulnerable to oxidative stress due to its high consumption of oxygen and glucose, enrichment in unsaturated fatty acids that are subject to oxidation, and presence of regions enriched in iron and ascorbate that are potent pro-oxidants for brain membranes. Moreover, higher levels of glucose upregulate the neuroinflammatory process measured as induction of iNOS and NO production [38]. Coupled with the relatively reduced antioxidant defenses in the brain, exposure of brain cells to reactive oxygen or nitrogen species can be detrimental and is thought to contribute to the pathogenesis of many brain disorders [39]. Oxidative stress is important in the etiology of EAE and is thought to contribute directly to CNS damage [7,40]. *N*-acetyl-L-cysteine (NAC) as cysteine, a precursor of glutathione, is a potent anti-oxidant. By scavenging superoxide radicals, metallothionein and other antioxidants such as cysteine, *N*-acetyl-cysteine and glutathione offer neuroprotection [41]. *In vivo *NAC enhances hippocampal neuronal survival after transient forebrain ischemia in rats [42]. Partial protection of neurons from the dopaminergic neurotoxin N-methyl-4-phenyl-1,2,3,6-tetrahydropyridine was achieved by four different antioxidants including NAC in the mouse [43]. NAC also has a protective effect in pneumococcal meningitis in the rat [44]. *In vitro*, NAC promotes oligodendrocyte survival in the presence of toxic stimuli or due to withdrawal of growth factors [45] and maturation of oligodendrocytes [46]. NAC inhibits Theiler's virus-induced NO and TNF-α production by murine SJL/J astrocyte cultures [47]. NAC treatment prevented cytokine-induced decrease in GSH and degradation of sphingomyelin to ceramide, also blocked cytokine-mediated ceramide production in rat primary oligodendrocytes, microglia, and C6 glial cells, thereby preventing cell death. These results suggest that intracellular levels of GSH may play a critical role in the regulation of cytokine-induced generation of ceramide leading to apoptosis of brain cells in demyelinating diseases. [48]

In summary, the ability of NAC to inhibit the induction of proinflammatory cytokines and inhibit the transmigration of inflammatory cells into the CNS of EAE-induced rats identifies it as a potential drug for the treatment of neuroinflammatory diseases and possibly other Th1-mediated autoimmune diseases. In addition, *in vitro *studies suggest that NAC may also promote survival of neurons and oligodendrocytes and thereby potentially facilitating remyelination. MS is a multifactorial disease and the etiology of the disease in unknown. Consequently, the targets for the prevention of the disease are currently unknown. However the disease signs and causes of these are known. For example an increase in pro-inflammatory cytokines and iNOS activity has been linked increase in clinical sign. As evidenced in the manuscript, NAC can inhibit the production of inflammatory cytokines and nitrotyrosine in the CNS during EAE pathogenesis. Thus, NAC holds out to be a promising therapeutic agent for the amelioration of MS/EAE.
